# Seroprevalence and Clinical Outcomes of *Neospora caninum*, *Toxoplasma gondii* and *Besnoitia besnoiti* Infections in Water Buffaloes (*Bubalus bubalis*)

**DOI:** 10.3390/ani10030532

**Published:** 2020-03-22

**Authors:** Lavinia Ciuca, Giuliano Borriello, Antonio Bosco, Luigi D’Andrea, Giuseppe Cringoli, Paolo Ciaramella, Maria Paola Maurelli, Antonio Di Loria, Laura Rinaldi, Jacopo Guccione

**Affiliations:** Department of Veterinary Medicine and Animal Production, University of Naples Federico II, Via Delpino 1, 80137 Naples, Italy; lavinia_vet1@yahoo.com (L.C.); giuliano.borriello@unina.it (G.B.); boscoant@tiscali.it (A.B.); giuseppe.cringoli@unina.it (G.C.); paociara@unina.it (P.C.); mariapaola.maurelli@unina.it (M.P.M.); adiloria@unina.it (A.D.L.); laura.rinaldi@unina.it (L.R.); jacopo.guccione@unina.it (J.G.)

**Keywords:** *Neospora caninum*, *Toxoplasma gondii*, *Besnoitia besnoiti*, water buffaloes, aborting pathogens, Italy

## Abstract

**Simple Summary:**

Over the recent years, increasing demand for buffalo products and consequently expanding its productivity has generated concerns regarding diseases that reduce fertility or cause abortion but the attention has been focused mostly on infectious diseases. Thus, exploration on the capacity of parasitic pathogens in relation to reproductive losses in this species are needed. This was the first study investigating, simultaneously, the role and changes induced by *Neospora caninum*, *Toxoplasma gondii* and *Besnoitia besnoiti* in water buffaloes in southern Italy. The outcome of this study revealed a high exposure of water buffaloes to both *N. caninum* and *T. gondii,* whereas all the animals resulted negative to *B. besnoiti*. The mono-infection with *N. caninum* seems mainly associated with abortion and presence of retained foetal membranes, while mono-infection with *T. gondii* has been associated with an increase of days open. In case of co-infections with both pathogens, the effects on the animals are related to abortion and embryonic death. The outcome of this study may be considered the starting point to promote the awareness about parasitic infections in buffalo medicine.

**Abstract:**

One hundred twenty-four water buffaloes (*Bubalus bubalis*) originating from 9 farms located in southern Italy were tested to investigate simultaneously, for the first time, the seroprevalence of the protozoa *Neospora caninum, Toxoplasma gondii* and *Besnoitia besnoiti* by ELISA tests and to evaluate the clinical findings potentially associated to the presence of these aborting parasitic pathogens. Twenty-five of 124 buffaloes (20.2%) were positive for *N. caninum*, while 17/124 (13.7%) for *T. gondii*. No buffalo showed specific antibodies for *B. besnoiti.* Nineteen of 124 animals (15.3%) were found seropositive for both *T. gondii* and *N. caninum*. The univariate statistical analysis showed that the seroprevalence of *N. caninum* is significantly associated with abortion and presence of retained foetal membranes, while the seroprevalence of *T. gondii* is significantly associated with an increase of days open. The logistic regression models showed that the co-infection by *N. caninum* and *T. gondii* strengthened the abortive effects (OR = 7.330) and showed further negative effects on the parameter embryonic death (OR = 2.607). The outcome revealed herein represents a high exposure of *N. caninum* and *T. gondii* in water buffaloes with reproduction disorders that deserves attention for both economic reasons, animal health and welfare.

## 1. Introduction

Water buffalo (*Bubalus bubalis*) breeding system represents an important Italian economic resource and mozzarella cheese manufacturing is third-ranked as agricultural economy concerning sales volume [1]. Whereas water buffalo medicine suffers from poor clinical scientific knowledge if compared to cattle [2,3,4], estimation under field conditions of clinical findings associated to parasitic infections is an interesting challenge in this species where knowledge regarding the health consequences of the most common pathologies as well as their economic impact on the entire dairy food-chain are still almost rare [5,6,7]. In the recent years, increasing demand for dairy products and consequent maximizing productivity of buffalo herds have raised concerns regarding diseases that lower fertility or cause abortion in this livestock species but the attention has been focused mostly on infectious diseases [8]. Thus, investigations on the implications of parasitic pathogens in relation to reproductive losses in this species are needed [9,10,11].

In addition to having in common a heterogeneous life cycle, the Apicomlexa protozoan parasites *Neospora caninum, Toxoplasma gondii, Besnoitia besnoiti* are responsible for negative impact on reproductive efficiency in farm ruminants worldwide [12,13,14].

The first of the three aborting pathogens, *N. caninum* can infect dogs and other canids as definitive hosts and is considered one of the most common causes of abortion in livestock as cattle, sheep, goats, water buffaloes and camels [14,15]. The seroprevalence of *N. caninum* in water buffaloes can greatly change either between countries or when the protozoan is cause of mono-infection rather than co-infection. Indeed, it can vary from the 24.3% reported in Mexico up to 66.7% described in Israel [15,16] and a recent investigation in northern Brazil showed a *N. caninum* seroprevalence of 44.0% but 14.8% of the infected animals were co-infected with *T. gondii* [17]. Studies performed in Italy showed a seroprevalence of *N. caninum* in water buffaloes ranging between 34.6% and 59% [18,19]. Similar results have been reported in cattle, equids and wild ruminants [20,21,22,23]; although any co-infections were investigated so far.

The second aborting pathogens, *T. gondii* can infect cats and other felids as definitive hosts, and is responsible for clinical disease in the intermediate host species, e.g., farm animals and humans [24,25,26]. *T. gondii* antibodies are less prevalent in cattle and buffaloes comparing with other members of the *Bovidae* family (e.g., sheep and goats), thus suggesting that large ruminants are more resistant to *T. gondii* [25]. The seroprevalence of toxoplasmosis in water buffaloes reared in several countries across the globe ranged between 15.2% and 20.4% in south Asia, 36.2% and 48.7% in Veracruz (Mexico), and levels of 14.3% are reached in South-West Iran [27,28,29]. Recently, a study from Romania, that has the second largest population of buffaloes in Europe, after Italy, reported a total of 2.7% prevalence of *T. gondii* in water buffaloes [30]. Unfortunately, no updated data are available on the seroprevalence of *T. gondii* in water buffaloes in Italy, and the last reliable data are outdated (94.0% of seroprevalence) [31]. Despite the lack of studies regarding clinical findings in water buffaloes affected by *T. gondii*, toxoplasmosis has been widely acknowledged as cause of abortion, stillbirth, fever, dyspnea and neurological signs in large ruminants [32].

As regards the third pathogen *B. besnoiti*, scarce information is available on its epidemiology and clinical impact on the infected animals. This parasite recognizes domestic cats as potential definitive hosts, and seems able to induce abortion, cutaneous and general disorders (e.g., tissue cysts, elephant skin, fever, hemorrhages) in both domestic and wild intermediate hosts [16,33,34]. In 2010, bovine besnoitiosis had been recognized as a European emerging disease as different cases were described in France, Spain, Italy and Germany [35]. In 2013, its geographical expansion to other European countries as well as its economic impact on livestock breeding systems significantly increased [36]. To the best of the authors’ knowledge, there are rare information regarding the prevalence and epidemiology of *B. besnoiti* infections in buffaloes [10,16,37] while no findings are reported about the clinical evidence of the infections in these ruminants. Nevertheless, a previous study performed on a population of cattle reared in southern Italy showed a seroprevalence of 44.1% against *B. besnoiti* [37].

Finally, there are few data regarding co-infections by these protozoa in water buffaloes worldwide and no information are available about the simultaneous evaluation of all three pathogens in water buffaloes in Europe.

Therefore, the aims of the present study were to investigate for the first time (i) the seroprevalence of *N. caninum, T. gondii* and *B. besnoiti* in water buffaloes in southern Italy and (ii) the clinical findings associated to the presence of these protozoa, in order to understand the consequence on water buffalo health of the exposure to the three pathogens, considering that *B. besnoiti* had never been investigated in these large ruminants in Europe.

## 2. Materials and Methods

### 2.1. Farms and Animals

This study was carried out between May and July 2017, on 124 water buffaloes reared in 9 intensive dairy farms located in southern Italy (Campania region). Farms were selected following convenient sampling by the local veterinary surgeon specialist in water buffalo reproduction and responsible for the farms. All the farms were characterized both by a spring-summer deseasonalized calving system and by barns with solid grooved concrete floors in the walking and feeding alleys. The lying area consisted either of elevated cubicles covered with rubber mattresses for milking buffalo calves or of a roofed deep straw yard area for dry water buffaloes. Presence of stray and/or owned dogs and cats could not be excluded in all the farms and none of them were performing a routine clinical-parasitological monitoring program at examination time. 

Criteria of eligibility for the water buffaloes were: (1) selected for the slaughterhouse because of poor reproductive performance targets (≥600 Days Open (DO)); (2) absence of macroscopic reproductive disorders potentially responsible of poor fertility performances. All the enrolled animals had been tested by the governmental office for veterinary public health for brucellosis (*Brucella abortus*) and tuberculosis (*Mycobacterium bovis*) and resulted negative.

The current investigation received an institutional approval by the Ethical Animal Care and Use Committee of the University of Naples Federico II (n.PG/2017/0099607), moreover all the farmers involved were previously informed and agreed about the purpose and methods used.

### 2.2. Clinical Procedures and Serological Diagnosis

For each animal enrolled, a history regarding clinical and reproductive parameters was collected from the farms’ databases. Detailed definitions of clinical status considered are reported in Table 1. Briefly, the following information on clinical-reproductive parameters were collected: number of Days Open (No. DO), presence or absence of abortion (AB), number of abortions (No. AB), embryonic death (ED), number of embryonic deaths (No. ED), presence or absence of retained foetal membranes (RFM) and total number of retained foetal membranes (No. RFM); the age of the animals was also recorded. Each water buffalo enrolled was submitted to a complete clinical examination with particular focus on the reproductive apparatus performed through visual examination of external genitalia, trans-rectal palpation and ultrasound according to the indication of Bond et al. [38] (data not shown). Jugular blood was collected in tube containing serum separator (Vacutainer^®^, Becton and Dickinson, Franklin Lakes, NJ, USA) at time of examination. Immediately after blood collection, serum was extracted by means of centrifugation (908 g × 15 min) and stored at −20 °C until serological screening (after two weeks) for the three protozoa pathogens.

The sera samples were analyzed by three indirect enzyme-linked immunosorbant assays (ELISAs). Indirect ELISA kits (ID.Vet, France, Indirect Toxoplasmosis Multispecies; ID.Vet, France, Indirect *Neospora caninum* for ruminants; ID.Vet, France Indirect *Besnoitia besnoiti* for cattle) were used according to the manufacturer’s instructions to detect anti-*T. gondii*, anti-*N. caninum* and anti-*B. besnoiti* specific antibodies in water buffaloes. Each sample was tested in duplicate. The optical density (OD) was measured at 450 nm in an Multiskan Go (Thermo, Naples, Italy). The S/P percentage of samples was calculated as follows: (mean OD value of the sample – mean OD value of negative control)/(mean OD of positive control – OD value of negative control) multiplied by 100. Samples with an S/P value ≥ 50% were considered to be seropositive for toxoplasmosis and for neosporosis. The S/P percentage of samples for besnoitiosis was calculated as follows: (mean OD value of the sample/mean OD value of positive control) multiplied by 100. S/P value ≥ 30% were considered to be seropositive.

### 2.3. Statistical Analysis

All the data (age and clinical-reproductive parameters) were analyzed by univariate Pearson’s Chi-square test for independence. Animals were divided into four age groups (<5 years; 5–10 years; 10–15 years; >15 years) and into four categories based on number of DO (<705 days; 706–725 days; 726–750 days; <750 days). Moreover, the animals were divided by number of abortion (No. AB = 0, 1, 2); by number of embryonic deaths (No. ED = 0, 1, 2, 3) and by number of retained foetal membranes (No. RFM = 1, 2). Subsequently, multivariate logistic regression models were used to identify the most important risk factors for *N. caninum* seropositivity and *T. gondii* seropositivity. Each model was applied at buffalo individual level, using all the data (age and clinical-reproductive parameters) recorded, as independent variables and the *N. caninum* or *T. gondii* (positive/negative) and mixed infection (total positive/total negative) serological status, as dependent variables. If interaction between variables was suspected, logistic regression models were run with and without these variables in order to evaluate possible effect modification on their behalf [41]. The odds ratio (OR) was used to estimate the strength of the association between each clinical parameter included in the study and the positive status to *N. caninum* and *T. gondii.* The independent variables considered in the final model were those showing *P*robabilities < 0.05. All the statistical data were analyzed using dedicated software (SPSS, Version 17, Chicago, IL, USA).

## 3. Results 

### 3.1. Clinical and Parasitological Findings 

Clinical-reproductive parameters data of the enrolled animals are reported in Table 2. All the farms resulted negative for *B. besnoiti* but positive for at least one of the two other protozoa investigated. Indeed, 7 farms (78%, 95% CI = 45–94) had buffaloes co-infected by both *N. caninum* and *T. gondii*. Instead, 1 farm (11%, 95% CI = 2–43) had buffaloes positive only for *T. gondii*. 

Overall a total of 61/124 (49.2% 95%CI = 40.2–58.3) water buffaloes were categorized as antibody positive for at least one of two aborting protozoa considered, as follows: 25 of 124 animals (20.2%; 95%CI = 13.7–28.6) tested seropositive only for *N. caninum*, while 17 of 124 (13.7%, 95%CI = 8.4–21.3) only for *T. gondii.* Nineteen of 124 animals (15.3%, 95%CI = 9.7–23.2) had resulted seropositive to both protozoa (*T. gondii* and *N. caninum*).

### 3.2. Univariate and Multivariate Logistic Regression 

The results of the univariate statistical analysis are reported in Table 2. Briefly, although mono-infection with either *N. caninum* or *T. gondii* was not associated with the age parameter (*p* > 0.05), the highest *N. caninum* seroprevalence (25.0%) was found in buffaloes <5 years old while the highest one for *T. gondii* (33.3%) was detected in animals >15 years old. No significant association (*p* > 0.05) was found between mono-infection with *N. caninum* or *T. gondii* and clinical-reproductive parameters as ED, No. ED, RFM, No. RFM. A statistically significant association (*p* < 0.05) was instead observed between buffaloes mono-infected with *T. gondii* and No. DO (No. DO < 705). A significant difference (*p* < 0.05) with a strong correlation (Gamma = 0.685) [42 was observed between the presence of *N. caninum* and aborting water buffaloes as well as with the number of abortions (*p* < 0.05, Gamma = 0.699) [42].

The results of the logistic regression models (Table 3) showed a positive association between the seropositivity for *N. caninum* and the following parameters: presence of AB (OR = 5.641; 95% CI: 2.136–14.893) and No. of RFM (OR = 2.095; 95% CI = 1.057–7.980). No association was found between the seropositivity to *T. gondii* and any of the clinical-reproductive parameters. 

Regarding the co-infection two models were created; the first showed an association between the seropositivity of mixed infection (*N. caninum* + *T. gondii*) with abortion (OR = 7.330; 95%CI = 3.037–17.690) and seropositivity to *T. gondii* (OR = 4.054; 95%CI = 1.592–10.323). An association between mixed infection (i.e., *T. gondii* + *N. caninum* seropositivity) with embryonic death (OR = 2.607; 95%CI = 1.137–5.978) and seropositivity to *N. caninum* (OR = 2.992; 95%CI = 1.309–6.840) was detected in the second model.

## 4. Discussion 

Estimation under field conditions of seroprevalence and clinical findings associated with *N. caninum, T. gondii* and *B. besnoiti* infections is an interesting challenge in water buffalo medicine where the knowledge regarding these parasites are almost rare [9,10,31]. Indeed, to the best of the authors’ knowledge, this was the first study investigating, simultaneously the role and changes induced by these pathogens in water buffaloes, since the knowledge regarding *N. caninum and T. gondii* on buffalo health are truly incomplete, while those relative to *B. besnoiti* are completely absent in Europe.

Regarding *N. caninum*, our data indicate a seroprevalence of 20.2%, almost half of what described by Reichel et al. [43]. In their review, the authors indicated the mean seroprevalence of neosporosis for the buffalo populations across the globe as approximately 3 times higher (48.4%) than those observed in cattle, suggesting water buffalo as more susceptible to subclinical infection than cow. Moreover, our results showed an association between the response of the animals to *N. caninum* antibodies with the aborting buffaloes as well as with RFM presence. Either regarding the observed seroprevalence or the clinical findings, our results seem also to confirm what described both by Guarino et al. [18] and by Auriemma et al. [19]. Indeed, both the studies described the presence of the parasite in water buffaloes in our own study area (southern Italy) suggesting, at the same time, an important role of *N. caninum* as a possible abortion pathogen. During our investigation *N. caninum* seems to confirm its abortive properties either when in mono-infection or in co-infection (*N. caninum* + *T. gondii).* Its pathogenic role has been already widely demonstrated in buffalo by Auriemma et al. [19]. Indeed, according to their results a causal relationship between *N. caninum* presence and lesions at brain and heart level of the same animals has been established in fetuses aborted in mid-gestation. To the best of the author’s knowledge, it is instead the first time that RFM has been associated to *N. caninum* infection. In 2015, Chryssafidis et al. [44] described the lesions in foetal membranes of buffaloes infected by *N. caninum* in an experimental study, but they did not observe this pathologic status. The association between *N. caninum* infection and RFM is instead widely known in dairy cattle where has been demonstrated how the tachyzoite invasion of feto-maternal interface produce a status of inflammation/necrosis inhibiting the post-parturient macrophage activity necessary for a normal foetal membranes release [45,46]. A similar ethology may be supposed also for buffalo but the assumption needs further and more detailed pathogenetic investigation before to be confirmed.

Since a long time, it is known that buffaloes are instead considerably resistant to *T. gondii* infection, nevertheless no recent studies exploring presence, role and clinical effects of this parasite in Italian buffaloes are reported in literature [47]. Herein, the presence of antibodies for *T. gondii* was reported in all the farms included in the present study although associated with a bit lower prevalence of positive animals than what observed for *N. caninum* (20.2% vs. 13.7%). The results seem to confirm what pointed out by da Silva et al. [17] in a different buffalo breed and reporting similar prevalence of these two parasites. According to the results outlined in the present investigation, the presence of this parasite has been associated for the first time with a high number of DO in positive buffaloes. *T. gondii* is not a common agent of abortion in cattle [48], as opposed to what reported for sheep and goats where the parasite can be considered one of the major causes of neonatal mortality and abortion [49,50]. Nevertheless, only in sheep it has been demonstrated that the infection in early pregnancy (less than 60 days), before the fetus acquires immunological competence, usually results in embryonic death and resorption. Although a similar effect may explain the abnormalities observed in water buffaloes affected by mono-infection (high number of DO) and in co-infection (*T. gondii + N. caninum,* presence of ED) due to *T. gondii*, this interesting hypothesis needs further investigations to avoid a potential overestimation of the results observed. For these reasons, a future study should be conducted to assess the effects of *T. gondii* in a greater sample population, excluding other physiological (e.g., seasonality) or pathological (e.g., uterine infections) factors negatively influencing the DO parameter.

Either *N. caninum* or *T. gondii* showed an overall high prevalence in the buffaloes enrolled. As reported for cows, the high prevalence to both pathogens may be explained by the mean age of the positive animals (older animals are more exposed to oocysts than younger) as well as by the presence of canids and felids in the enrolled farms (risk factor for both the infections ensuring their persistence in the farms) [49,50,51]. Based on the overall results of the study and the clinical outcomes detected, *N. caninum* and *T. gondii* should be considered in the routine diagnosis of abortive agents in water buffaloes, especially in herds characterized by poor fertility performances or pregnancy losses.

Lastly, some clinical comments regarding the absence of specific antibodies for *B. besnoiti* in the water buffaloes sampled. To the author’s knowledge, there are few information about the presence and the clinical effect of *B. besnoiti* in water buffaloes from Egypt [10] and Israel [16] even though it is a well-known pathogen of other bovids [33] and has been reported in cattle in southern Italy [37]. The results of our study may suggest that water buffaloes are less susceptible to *B. besnoiti* infection than cows, but the authors cannot completely exclude the sensitivity to the parasite of these dairy ruminants. Further studies associated with a wider sample population should be performed to verify the accuracy of the hypothesis.

However, the outcome revealed herein, represent an important exposure to *N. caninum* and *T. gondii* in buffaloes with reproduction disorders that deserves attention for both economic reasons, animal welfare and the risk to public health.

One limitation of our study could be due to the indirect diagnosis of the three protozoa by ELISA tests that may show some level of cross-reactivity [9]. The sera samples were analyzed by three commercial ELISA kits that are either multispecies or used for small and large ruminants. Although there are no validation data on the use of these commercial ELISA kits for serological detection of *Neospora, Toxoplasma* and *Besnoitia* infections in water buffaloes, previous studies have been already published on serological diagnosis of the pathogens mentioned above using these tests for either cattle or water buffaloes [52,53]. Indeed, the ELISA test that we used in the present study showed high sensitivity and specificity values (>95%) for the serological diagnosis of bovine neosporosis in a previous study conducted by Alvarez-Garcia et al. (2013) [53]. Moreover, the same ELISA test that was used in the present study for diagnosing besnoitiosis in water buffaloes, showed a 97.2% sensitivity and 100% specificity values for diagnosis of besnoitiosis in bovines in a study performed by Garcia-Lunar et al. [52]. However, another study reported a lower seroprevalence of antibodies to *T. gondii* in buffaloes and bovines using a different commercial ELISA test kit (Institut Pourquier, Montpellier, France) [49] and this kit was previously used by Vesco et al. [54] to conduct a large-scale serological survey of *T. gondii* in Italian sheep.

## 5. Conclusions

The outcomes revealed herein represent a high exposure of *N. caninum* and *T. gondii* in water buffaloes with reproduction disorders that deserves attention for issues related to economic reasons, animal health and welfare. The mono-infection with *N. caninum* seems mainly associated with abortion and presence of retained foetal membranes, while mono-infection with *T. gondii* has been associated with an increase of days open. In case of co-infections with both pathogens, the effects on the animals are related to abortion and embryonic death. The outcome of this study may be considered the starting point to promote the awareness about parasitic infections in buffalo medicine. Our results could be useful for improving parasitic disease control in water buffaloes.

Even though the clinical screening of the two parasites may be suggested in the routine diagnosis of abortive agents in water buffalo herds characterized by poor fertility performances or pregnancy losses, the assessment of their clinical effects on a larger sample population, as well as the in-depth analysis of the infectious dynamics, warrant further scientific attention with the goal to fully understand their pathogenic role in these animals.

## Figures and Tables

**Table 1:** Definition of the historical parameters collected for the water buffaloes enrolled during the investigation according to Hudson et al. [39] and Toni et al. [40].

Status	Definition
DO	Average number of days from calving to conception
AB	Expulsion of a recognizable dead/non-viable fetus prior the end of normal gestation
No. AB	Number of abortions
ED	Loss of conceptus within 45 days of pregnancy
No. ED	Number of embryonic deaths
RFM	Retention of foetal membranes for more than 24 h after calving

DO: Days Open; AB: abortion; No: number; ED: embryonic death; RFM: retained foetal membranes.

**Table 2:** Seroprevalences of *N. caninum*, *T. gondii* and mixed infection (*N. caninum* and *T. gondii*) in buffaloes based on age categories and the clinical-reproductive parameters.

Age Categories and Clinical-Reproductive Parameters	No. Tested Buffaloes	Serological Results: no. Positive, (%), (95% CI)		
		*N. caninum*	*T. gondii*	Mixed Infection
**Age categories**				
<5 years	28	7 (25.0%) (11.4–45.2)	3 (10.7%) (2.8–29.3)	4 (14.3%) (4.6–33.5)
5–10 years	69	12 (17.4%) (9.6–28.8)	7 (10.1%) (4.5–20.3)	11 (15.9%) (8.6–27.1)
10–15 years	21	5 (23.8%) (9.1–47.5)	5 (23.8%) (9.1–47.5)	4 (19.0%) (6.2–42.5)
>15 years	6	1 (16.7%) (0.8–63.5)	2 (33.3%) (6.0–75.8)	0 (0%) (1.6–48.3)
**Clinical-reproductive parameters**				
**Presence of Abortion (AB)**				
Yes	49	18 (36.7%) * (23.7–51.7)	2 (4.1%) (0.7–15.1)	11 (22.5%) (12.3–36.9)
No	75	7 (9.3%) (4.1–18.8)	15 (20%) (11.9–31.2)	8 (10.7%) (5.1–20.5)
**Number of AB**				
1	47	17 (36.8%) ** (23.0–51.5)	2 (4.3%) (0.7–15.8)	10 (21.3%) (11.2–36.1)
2	2	1 (50%) (2.6–97.3)	0 (0%) (4.9–80.2)	1 (50%) (2.7–97.3)
**Embryonic death (ED)**				
Yes	60	8 (13.3%) (6.3–25.1)	10 (16.75) (8.7–28.9)	13 (21.7%) (12.5–34.5)
No	64	17 (26.6%) (26.6–39.3)	7 (10.9%) (4.9–21.8)	6 (9.4%) (3.9–19.9)
**Number of ED**				
1	43	7 (16.3%) (7.3–31.3)	9 (20.9%) (10.6–36.5)	8 (18.6%) (8.9–33.9)
2	15	1 (6.7%) (0.4–33.9)	1 (6.7%) (0.4–33.9)	4 (26.7%) (8.9–55.2)
3	2	0 (0%) (4.9–80.2)	0 (0%) (4.9–80.2)	1 (50%) (2.7–97.3)
**Retained foetal membranes (RFM)**				
Yes	67	17 (25.4%) (15.9–37.4)	11 (16.4%) (8.9–27.9)	10 (14.9%) (7.8–26.2)
No	57	8 (14.0%) (6.7–26.4)	6 (10.5%) (4.4–22.2)	9 (15.8%) (7.9–28.4)
**Number of RFM**				
1	62	17 (27.4%) (17.2–40.4)	10 (16.1%) (8.4–28.1)	9 (14. 5%) (7.3–26.3)
2	5	0 (0%) (1.9–53.7)	1 (20%) (1.1–70.1)	1 (20%) (1.1–70.1)
**Number of Days Open categories (No. DO)**				
<705	32	7 (21.9%) (9.9–40.4)	9 (28.1%) *** (14.4–46.9)	3 (9.4%) (2.5–26.2)
706–725	32	8 (25%) (12.1–43.8)	3 (9.4%) (2.5–26.2)	2 (6.3%) (1.1–22.2)
726–750	33	6 (18.2%) (7.6–36.0)	2 (6.1%) (1.1–21.6)	9 (27.3%) (13.9–47.8)
>750	27	4 (14.8%) (4.9–34.6)	3 (11.1%) (2.9–30.3)	5 (18.5%) (7.0–38.8)
Totals	124	25 (20.2%) (13.7–28.6)	17 (13.7%) (8.4–21.3)	19 (15.3%) (9.7–23.2)

* Indicates significant difference: chi-square test = 13.824, *p* < 0.000; ** chi-square test = 14.052, *p* < 0.000; *** chi-square test = 7.915, *p* < 0.048.

**Table 3:** Results of logistic regression model ^a^.

Clinical-Reproductive Parameters and Age (Independent Variable)	Standard Error	*p*-Values	Odds Ratio	95 % Confidence Interval	
				Inferior	Superior
**Model 1 ^b^**					
Abortion	0.495	0.000	5.641	2.136	14.8 93
No. RFM (categ. 1)	0.516	0.039	2.095	1.057	7.980
**Model2 ^c^**					
Abortion	0.450	0.000	7.330	3.037	17.690
*Toxoplasma* seropositivity	0.477	0.003	4.054	1.592	10.323
**Model 3 ^d^**					
*Neospora* seropositivity	0.422	0.009	2.992	1.309	6.840
ED	0.423	0.024	2.607	1.137	5.978

^a^ Significant association between *N. caninum* and *T. gondii* seropositivity and clinical-reproductive parameters and age. ^b^ Dependent variable is the *N. caninum* seropositivity. ^c^ Dependent variable is the mixed infection Neo_Toxo seropositivity. ^d^ Dependent variable is the mixed infection Toxo_Neo seropositivity.
